# Inhibition of MDM2 via Nutlin-3A: A Potential Therapeutic Approach for Pleural Mesotheliomas with MDM2-Induced Inactivation of Wild-Type P53

**DOI:** 10.1155/2018/1986982

**Published:** 2018-07-17

**Authors:** Robert F. H. Walter, Robert Werner, Michael Wessolly, Elena Mairinger, Sabrina Borchert, Jan Schmeller, Jens Kollmeier, Thomas Mairinger, Thomas Hager, Agnes Bankfalvi, Daniel C. Christoph, Wilfried E. E. Eberhardt, Till Plönes, Clemens Aigner, Kurt W. Schmid, Jeremias Wohlschlaeger, Fabian D. Mairinger

**Affiliations:** ^1^Institute of Pathology, University Hospital Essen, University of Duisburg-Essen, Essen, Germany; ^2^Ruhrlandklinik, West German Lung Center, University Hospital Essen, University of Duisburg-Essen, Germany; ^3^Department of Pathology, Helios Klinikum Emil von Behring, Berlin, Germany; ^4^Department of Pneumology, Helios Klinikum Emil von Behring, Berlin, Germany; ^5^Department of Medical Oncology, West German Cancer Center, University Hospital Essen, University of Duisburg-Essen, Essen, Germany; ^6^Department of Internistic Oncology, Kliniken Essen Mitte, Essen, Germany; ^7^Department of Thoracic Surgery and Thoracic Endoscopy, Ruhrlandklinik, University Hospital Essen, University of Duisburg-Essen, Essen, Germany; ^8^Department of Pathology, Diakonissenkrankenhaus Flensburg, Flensburg, Germany

## Abstract

Previously, our group demonstrated that nuclear expression of E3 ubiquitin ligase (MDM2) in malignant pleural mesothelioma (MPM) is significantly associated with decreased overall survival. A possible explanation may be that overexpression of MDM2 leads to a proteasomal degradation of TP53 that eventually results in a loss of TP53-induced apoptosis and senescence. It is well known from other tumor entities that restoration of TP53 activity, e.g., by MDM2 inhibition, results in an instant TP53-induced stress and/or DNA damage response of cancer cells. Nutlin-3A (a* cis*-imidazoline analogue) has been described as a potent and selective MDM2 inhibitor preventing MDM2-TP53-interaction by specific binding to the hydrophobic TP53-binding pocket of MDM2. In the present study, the effects of MDM2 inhibition in MPM via Nutlin-3A and standard platinum based chemotherapeutic agents were comparatively tested in three MPM cell lines (NCI-H2052, MSTO-211H, and NCI-H2452) showing different expression profiles of TP53, MDM2, and its physiological inhibitor of MDM2—P14/ARF. Our* in vitro* experiments on MPM cell lines revealed that Nutlin-3A in combination with cisplatin resulted in up to 9.75 times higher induction of senescence (p=0.0050) and up to 5 times higher apoptosis rate (p=0.0067) compared to the commonly applied cisplatin and pemetrexed regimens. Thus Nutlin-3A, a potent inhibitor of MDM2, is associated with a significant induction of senescence and apoptosis in MPM cell lines, making Nutlin-3A a promising substance for a targeted therapy in the subgroup of MPM showing MDM2 overexpression.

## 1. Introduction

Malignant mesothelioma is a highly aggressive tumor arising from mesothelial lined surfaces, mostly from the pleural cavities (malignant pleural mesothelioma, MPM) [1, 2]. When untreated, the median survival of patients is nine months [3–5]. MPM patients are negatively affected by mostly insufficient current treatment modalities consisting of platinum-containing regimes using cisplatin [6] or carboplatin [7–10] as first choice. Cisplatin treatment results in a response rate of merely 14% and a median survival of less than seven months [11]. Carboplatin resulted in similar response rates ranging from 6 to 16% [11, 12]. In clinical practice, the antifolate pemetrexed, as the only FDA-approved therapeutic for MPM, is used in combination with platin compounds [6–10].

Several studies have shown the efficacy of the evaluation of intratumoral expression of members of the folic acid metabolism for prediction of multitargeted antifolate therapy response in patients with different cancer entities but are discussed controversially [10, 13–28]. As platin-analoga are genotoxic compounds that induce DNA damage [29] leading to TP53 induced cell cycle arrest and apoptosis [30], it is basically conceivable that the DNA repair mechanism might be one of the keys associated with an impaired therapy response. As the identification of molecular properties shared by MPMs may help to overcome the poor treatment response observed, several studies addressed this question [11, 12, 27, 31–34]. However, the reasons for the rather poor efficacy of platinum compounds remain largely unknown.

Summing up, neither reliable predictive biomarkers nor individualized therapeutic concepts for MPM exist until now. Therefore, current guidelines emphasize the need of innovative and novel therapies [35].

Since mutations of the* TP53* gene are extremely rare in MPM [36–38], other mechanisms such as deletion of the locus or epigenetic alterations may contribute to inactivation of TP53 [36–38]. Overexpression of MDM2 in some tumor types can lead to a loss of TP53 regulatory function in cancer cells by its increased proteasomal degradation [39–44]. P14/ARF, the physiological inhibitor of MDM2, is recognized as a tumor suppressor and contributes to this mechanism by induction of cell cycle arrest in both a TP53-dependent and TP53-independent manner. Moreover, miRNA regulation seems to play an important role [45–52]. In previous studies, we have demonstrated a strong nuclear MDM2 overexpression in approximately 25% of MPM; this observation was restricted to epithelioid MPM or the epithelioid components of biphasic MPM [44, 53]. Patients with MDM2-positive MPM showed a significantly decreased overall survival (OS) and progression-free survival (PFS) compared to MDM2-negative MPM [44, 53]. This might be explained by a significantly decreased or completely abolished TP53 activity and/or stability mediated by an overexpression of MDM2 [39–43].

A restoration of TP53 activity, e.g., by MDM2 inhibition, might result in an instant TP53 induced stress and/or DNA damage response of cancer cells. Nutlin-3A (a* cis*-imidazoline analogue) is a potent and selective MDM2 inhibitor with an IC_50_ value of 90nM [54] and prevents MDM2-TP53-interaction by binding to the hydrophobic TP53-binding pocket of MDM2 [55].

Thus, the aim of this study was to test the effect of MDM2 inhibition in MPM via Nutlin-3A in comparison to the contemporary common chemotherapeutic strategies using three cell lines showing different marker profiles concerning TP53-status, P14/ARF- and MDM2 expression level.

## 2. Material and Methods

### 2.1. Cell Line Experiments

Based on reviewing the literature, concentrations for the cytostatics were estimated (Nutlin-3A [55, 56], cisplatin [57], and pemetrexed [57], respectively).

Human MPM cell lines were obtained from the American Type Culture Collection in 2012-08 (Manassas, VA, USA). The cell lines were authenticated and tested for contaminations by using a commercial service (Multiplexion, Heidelberg, Germany) and were last retested directly after the experiments were finished.

NCI-H2052, NCI-H2452, and MSTO-211H were cultured in Roswell Park Memorial Institute (RPMI) medium (Invitrogen, CA, USA) containing 10% fetal bovine serum (Invitrogen) at 37°C in a 5% CO_2_-humidified atmosphere. Cells were grown until 85% to 95% confluency, then washed with phosphate-buffered saline (Invitrogen), and trypsinized with 1 ml of 0.05% trypsin-0.53 mM ethylenediaminetetraacetic acid, phenol red (Invitrogen). Trypsinization was stopped by adding fresh medium to the reaction. Approximately 10 *μ*l was transferred to a hemocytometer (BRAND, Wertheim, Germany) for cell counting purposes. 1,000 cells per well (100 *μ*l) were seeded into microplates 96/U (Eppendorf, Hamburg, Germany) suitable for luminescence and fluorescence detection. The cells were allowed to attach overnight at 37°C and 5% CO_2_. At the next day, the medium was removed and fresh medium containing either one of the cytostatics or without additive was applied to each well. Cisplatin (10*μ*M; TEVA, Petah Tikva, Israel) pemetrexed (200*μ*M; Lilly, IN, USA) and Nutlin-3A (5, 10 or 20*μ*M; Sigma-Aldrich, MO, USA) was applied either alone or in combination. Nutlin-3A had to be solubilized in dimethyl sulfoxide (Sigma-Aldrich). Concentrations of the applied cytostatics are summarized in Table 1. Cell cultures containing cytostatics and blank medium were incubated for three days at 37°C and 5% CO_2_. Within 72 hours, necrosis, apoptosis, and cell viability were assessed by using the following luminescence assays: CytoTox-Glo™ Cytotoxicity Assay (Promega), Caspase-Glo® 3/7 Assay (Promega), and CellTiter-Glo® Luminescent Cell Viability Assay (Promega). The assays were performed as recommended by the supplier. Per cytostatic drug and luminescence assay at least four data points were measured. Luminescence was assessed using a SpectraMax L Luminescence Microplate Reader (Molecular Devices, CA, USA). Luminescence (relative luminescent units; RLU) was measured at 570nm and integration time was adjusted to 1 second. Temperature of the SpectraMax L was kept between 21.5°C and 24.5°C during measurements. Additionally, from each cell line a FFPE block was prepared for immunohistochemical and qPCR analysis.

### 2.2. RNA Isolation and Real-Time qPCR

Expression levels of* ACTB* (reference gene),* MDM2* and* P14/ARF*, were investigated by TaqMan real-time qPCR in the three MPM cell lines. Therefore, RNA was isolated by cutting three to five sections of 4*μ*m from the FFPE block using a microtome (Leica, SM 2000 R, Wetzlar, Germany). Total RNA was isolated using the miRNeasy FFPE kit (Qiagen, Hilden, Germany) and manufacturer's protocol, except for two modifications (proteinase K digestion overnight; elution in 25*μ*l). RNA concentrations were measured using UV/VIS spectrometry (NanoDrop ND-1000, PEQLAB Biotechnologie GmbH, Erlangen, Germany). RNA was stored at -80°C. For cDNA synthesis, the iScript Select cDNA Synthesis Kit and protocol (Bio-Rad Laboratories, Inc., CA, USA) was used with an input of 1*μ*g total RNA per reaction.

For real-time qPCR, the TaqMan Gene Expression Assays on Demand (AoD) for* ACTB* (Hs03023943_g1),* MDM2* (Hs01066942_m1), and* P14/ARF* (Hs99999189_m1) were used (Applied Biosystems®; CA, USA). The reaction volumes were modified by using 50% of the recommended total reaction volumes with 50 ng cDNA input. Each target was measured in triplicate. Ct-values of* P14/ARF* and* MDM2* were normalized to the mean values of* ACTB*. Real-time qPCR and data analysis were performed on a Roche LightCycler 480 II (Roche, Basel, Switzerland) and corresponding software. All real-time qPCR experiments were performed in accordance with the MIQE-guidelines [58].

### 2.3. Immunohistochemistry

Immunohistochemistry was performed according to standard protocols using an automated stainer (Ventana Discovery XT, Munich, Germany). After validation on reference tissues (liposarcoma for MDM2, pulmonary adenocarcinoma for TP53), the immunohistochemical investigations were performed with antibodies directed against MDM2 (clone IF2, Calbiochem, Darmstadt, Germany, dilution: 1:80) and TP53 (clone BP53-12, Zytomed, Berlin, Germany; dilution: 1:5000). Pretreatment for antigen retrieval was performed by heating in deionized water at pH 6 for 30 minutes. Protein expression was assessed using a four-stage IHC scoring system based on the percentage of tumor cell nuclei with a positive immunoreaction (Score 0: no signal; Score 1 (weak expression): 1-25%; Score 2 (moderate expression): 26-50%; Score 3 (strong expression): >50%).

### 2.4. Statistical Analysis

Statistical and graphical analyses were performed with the R statistical programming environment (v3.4.2).

For analysis between single groups, either the Wilcoxon Mann–Whitney rank sum test (non-parametric) or two-sided students t-test (parametric) was applied. For ordinal variables with more than two groups (luminescence signal differences between all treatment groups), either the Kruskal-Wallis test (non-parametric) or ANOVA (parametric) was used to detect group differences.

The level of statistical significance was defined as p<0.05.

## 3. Results

**Figure 1:**
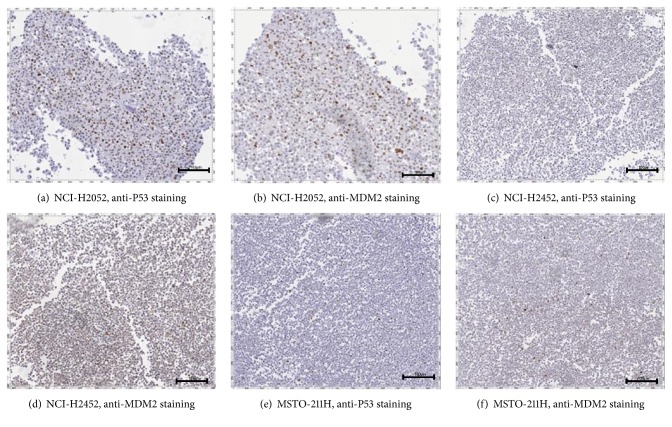
**Immunohistochemical staining of the investigated MPM cell lines with antibodies directed against P53 and MDM2**. NCI-H2052 shows a strong staining (Score 2) regarding P53 (a) and MDM2 (b). NCI-H2452 showed neither immunoexpression for P53 ((c), Score 0) nor for MDM2 ((d), Score 0). MSTO-211H stained positive for P53 ((e), Score 1) and MDM2 ((f), Score 1). The scale bars indicate 100 *μ*m for pictures (a) and (b) and 500 *μ*m for pictures (c), (d), (e) and (f).

**Figure 2:**
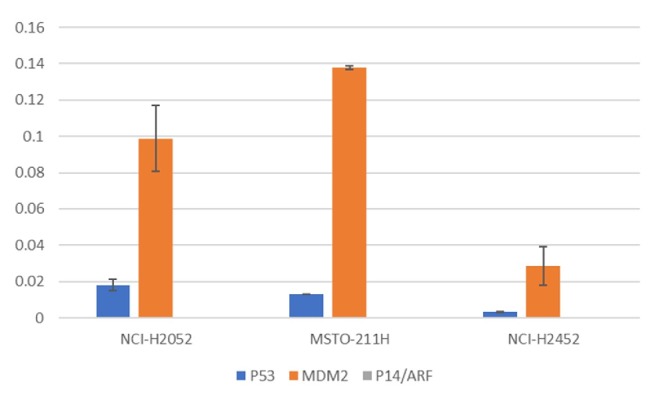
**The bar chart shows the relative mRNA expression of* MDM2*,* P53* and* P14/ARF* in the investigated MPM cell lines**. On the x-axis the investigated cell lines are shown and the respective mRNA expression of* P53*,* MDM2*, and* P14/ARF*. On the y-axis the 2∧ΔCt values for the relative mRNA expression of the investigated target genes is shown after normalization against the reference gene* ACTB *(actin, beta). NCI-H2052 and MSTO-211H show elevated expression of* MDM2*, whereas NCI-H2452 showed minimal* MDM2* expression.* TP53* mRNA expression was reduced in NCI-H2452 compared to both other cell lines.* P14/ARF* expression was below the detection limit in the investigated specimens.

The expression profiles of MDM2, TP53, and P14/ARF differ between the investigated cell lines and are summarized in Table 2. Scans of immunohistochemical staining's are shown in Figure 1; qPCR results are visualized in Figure 2. NCI-H2052 showed pronounced MDM2-immunoexpression, but little P14/ARF and TP53-expression. Immunohistochemically, MSTO-211H showed no expression of MDM2 and P14/ARF, but TP53-expression was present. NCI-H2452 showed neither MDM2- nor TP53-expression, but P14/ARF expression was detected. The investigated cell lines represent the molecular constellation that was reported in previous studies of patients with MPM [59, 60].

### 3.1. Response of MPM Cell Lines to Pemetrexed, Cisplatin, and Varying Nutlin-3A Concentrations

Cisplatin (10*μ*M) and pemetrexed (200*μ*M) as single agent as well as in combination were tested versus three Nutlin-3A concentrations (5*μ*M, 10*μ*M, and 20*μ*M).

#### 3.1.1. Cell Viability

*NCI-H2052. *Any Nutlin-3A concentration was superior in reducing cell viability compared to either cisplatin or pemetrexed or their combination, respectively (p=0.0039). In contrast, treatment with pemetrexed alone showed significantly elevated cell viability. Treatment with cisplatin alone showed higher cell viability than cisplatin and pemetrexed in combination.

*MSTO-211H. *Pemetrexed combined with cisplatin was associated with the highest cell viability, followed by cisplatin alone and the lowest Nutlin-3A concentration (p=0.0952). Pemetrexed combined with cisplatin reduced cell viability significantly, but Nutlin-3A (10*μ*M) exhibited a slightly stronger reduction. The highest Nutlin-3A concentration reduced cell viability to a minimum.

*NCI-H2452. *The highest Nutlin-3A concentration (20*μ*M) reduced cell viability to a minimum (p=0.0017). 10*μ*M Nutlin-3A was the second strongest cell viability inhibitor followed by cisplatin alone, pemetrexed alone, and cisplatin in combination with pemetrexed. The lowest Nutlin-3A concentration showed the weakest impact on cell viability reduction.

**Figure 3:**
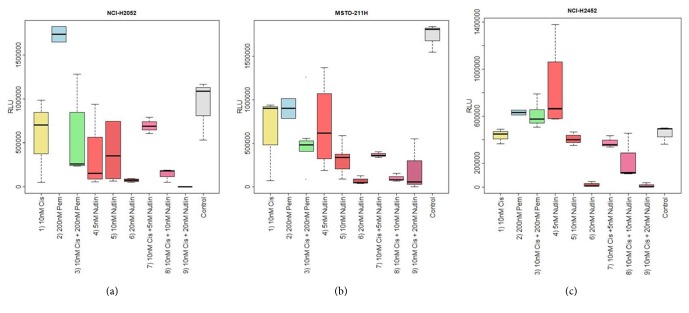
**Induction of senescence in MPM cell lines by pemetrexed, cisplatin, and varying Nutlin-3A concentrations as well as varying Nutlin-3A concentrations combined with cisplatin**.  Figure 3 shows boxplots for cell viability/senescence for the three investigated MPM cell lines. On the y-axis RLU (relative luminescence units) are shown. High RLU indicate high cell viability, whereas low RLU indicate senescence. On the x-axis, the concentrations of the cytostatics applied and the control are shown. In all three MPM cell lines, 20*μ*M Nutlin-3A showed the strongest inhibition of cell viability compared to the other single agent cytostatics and concentrations applied. This is true against other Nutlin-3A concentrations (NCI-H2052: p=0.021, MSTO-211H: p=0.007, NCI-H2452: p<0.001), cisplatin (NCI-H2052: p=0.021, MSTO-211H: p=0.018, NCI-H2452: p=0.004), pemetrexed (NCI-H2052: p=0.032, MSTO-211H: p=0.008, NCI-H2452: p=0.006), and a combination of both (NCI-H2052: p=0.003, MSTO-211H: p=0.002, NCI-H2452: p<0.001). Additionally, higher concentrations of Nutlin-3A (10*μ*M, 20*μ*M) combined with cisplatin regimen showed the strongest inhibition of cell viability compared to nowadays approved cytostatics, either as single agents (cisplatin: NCI-H2052: p=0.021, MSTO-211H: p=0.022, NCI-H2452: p=0.006; pemetrexed: NCI-H2052: p=0.014, MSTO-211H: p=0.029, NCI-H2452: p<0.001) or in combination (NCI-H2052: p=0.003, MSTO-211H: p=0.014, NCI-H2452: p<0.001).

Box plots for cell viability highlight decreasing cell viability with increasing Nutlin-3A concentration in the tested cell lines. The results for all cell lines regarding senescence/cell viability are summarized in Figures 3(a)–3(c).

#### 3.1.2. Apoptosis

*NCI-H2052. *In the NCI-H2052 cell line, the highest apoptosis rate was found for 20*μ*M Nutlin-3A, whereas the other treatment approaches showed similar apoptosis induction (p=0.14).

*MSTO-211H. *In MSTO-211H, highest apoptosis rates were found for pemetrexed followed by pemetrexed in combination with cisplatin and different Nutlin-3A concentrations (p=0.0219). Almost no apoptosis was observed for cisplatin alone and Nutlin-3A.

*NCI-H2452. *NCI-H2452 revealed the highest apoptosis rate in response to Nutlin-3A in the highest concentration (20*μ*M) followed by cisplatin (p=0.0359). Significantly lower apoptosis rates were found for the remaining cytostatics.

**Figure 4:**
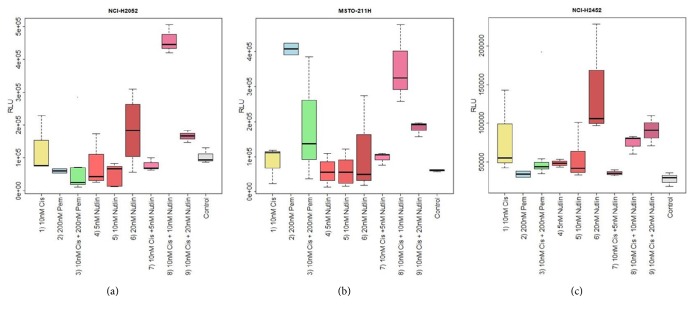
Figure 4 shows boxplots for apoptosis for the investigated MPM cell lines. On the y-axis RLU (relative luminescence units) are shown. RLU and increasing apoptosis rates show a direct correlation. On the X-axis, the concentrations of the cytostatics applied and the control are shown. For cell line NCI-H2052 and NCI-H2452 (shown in Figures 4(a) and 4(c), respectively), 20*μ*M Nutlin-3A showed the strongest induction of apoptosis when comparing single agents (cisplatin: NCI-H2052: p=0.084, NCI-H2452: p=0.028; pemetrexed: NCI-H2052: p=0.011, NCI-H2452: p=0.049) but also against cisplatin combined with pemetrexed (NCI-H2052: p=0.015, NCI-H2452: p=0.008). MSTO-211H (shown in Figure 4(b)), the highest apoptosis rate, was found for pemetrexed followed by 5*μ*M, 20*μ*M Nutlin-3A, and the combination of pemetrexed and cisplatin (all p<0.001). When analyzing Nutlin-3A in combination with cisplatin, for cell line NCI-H2052, (a) the highest apoptosis rate was found for 10*μ*M Nutlin-3A combined with cisplatin (all p<0.001). MSTO-211H (b) apoptotic rates of 10*μ*M Nutlin-3A combined with cisplatin comparable to the treatment with pemetrexed alone (p=0.493) significantly enhanced against all other approaches (p=0.016). In NCI-H2452 (c), treatment with cisplatin combined with 20*μ*M and 10*μ*M Nutlin3A showed the strongest induction of apoptosis beside 20*μ*M Nutlin-3A alone (p=0.004) but shows no statistically significant differences compared with 20*μ*M Nutlin-3A single agent treatment (p=0.199).

The results for apoptosis are summarized in Figures 4(a)–4(c).

#### 3.1.3. Necrosis

Necrosis of cells was not influenced by any of the chemotherapeutics compared to the control (data not shown).

### 3.2. Response of MPM Cell Lines to Varying Nutlin-3A Concentrations Combined with Cisplatin

In further experiments, the induction of apoptosis was tested by using either a Nutlin-3A regimen or a combination of Nutlin-3A and cisplatin. Three combinations of Nutlin-3A (5*μ*M, 10*μ*M, and 20*μ*M) plus cisplatin (10*μ*M) were compared with cisplatin (10*μ*M) alone, pemetrexed (200*μ*M) alone, Nutlin-3A alone (10*μ*M), and a combination of cisplatin and pemetrexed.

#### 3.2.1. Cell Viability

*NCI-H2052. *Nutlin-3A alone and its combination with cisplatin showed significantly increased induction of senescence compared to the other regimen (p=0.0051). Only 5*μ*M Nutlin-3A in combination with cisplatin showed lower potency to induce senescence rates as 5*μ*M Nutlin-3A without cisplatin. The higher Nutlin-3A concentrations (10 and 20*μ*M) with cisplatin reduced cell viability to a minimum. The highest cell viability was found for pemetrexed followed by the combination of pemetrexed and cisplatin.

*MSTO-211H. *Any combination of Nutlin-3A with cisplatin induced significantly increased cellular senescence compared to cisplatin, pemetrexed, or a combination of both (p=0.0059). However, the combination of cisplatin and pemetrexed showed similar efficacy compared to the lowest Nutlin-3A/cisplatin regimen and Nutlin-3A alone. Higher concentrations of Nutlin-3A combined with cisplatin reduced cell viability to a minimum.

*NCI-H2452. *Nutlin-3A in combination with cisplatin or alone was superior compared to the other cytostatics, except at the lowest concentration of 5*μ*M (p=0.0089). Interestingly, cisplatin showed comparable efficacy as 10*μ*M Nutlin-3A alone and cisplatin in combination with 5*μ*M Nutlin-3A. The highest cell viability was observed with pemetrexed, cisplatin in combination with pemetrexed, and 5*μ*M Nutlin-3A. The highest Nutlin-3A concentration (20*μ*M) with cisplatin showed the highest senescence rate.

Box plots for cell viability highlight that cell viability decreased with increasing concentration of the cisplatin/Nutlin-3A regimen in the tested cell lines. The results for senescence/cell viability are summarized in Figures 3(a)–3(c).

#### 3.2.2. Apoptosis

*NCI-H2052. *In the NCI-H2052 cell line, higher Nutlin-3A concentrations combined with cisplatin applied induced significantly increased apoptosis compared to pemetrexed alone or combined with cisplatin (p=0.0069). The highest apoptosis rates were found for 10*μ*M Nutlin-3A in combination with cisplatin. 

*MSTO-211H. *Cell line MSTO-211H exhibited the highest apoptosis when treated with pemetrexed alone (p=0.0035). The second highest apoptosis rate was found for 10*μ*M Nutlin-3A combined with cisplatin. Pemetrexed in combination with cisplatin resulted in the third highest apoptosis rate. Cisplatin in combination with 20*μ*M Nutlin-3A was more potent than cisplatin alone, Nutlin-3A alone, and the lowest concentration of Nutlin-3A (5*μ*M) in combination with cisplatin.

*NCI-H2452. *The highest apoptosis rates were found for the 20*μ*M Nutlin-3A single agent as well as 10*μ*M and 20*μ*M Nutlin-3A concentrations combined with cisplatin, followed by cisplatin (p=0.1).

The results regarding apoptosis are summarized in Figures 4(a)–4(c).

#### 3.2.3. Necrosis

Necrosis of cells was not influenced by any of the chemotherapeutics compared to the non-treated control (data not shown).

All results of the cell line inhibition experiments are summarized in Table 3.

## 4. Discussion

In previous studies we identified MDM2 as a prognostic biomarker in patients with MPM and that expression is regulated through specific miRNA [44, 52, 59]. Nutlin-3A inhibits MDM2-TP53 interaction and thereby induces cell cycle arrest, senescence, and apoptosis depending on the cell type [61, 62]. Additionally, it is a nongenotoxic drug that exhibits little toxicity in animal models and is associated with a lower risk of resistance than conventional drugs [61–63].

Against this background we hypothesized that MDM2 overexpression, maybe in combination with partial or complete loss of P14/ARF, can be targeted by a Nutlin-3A based therapy regimen to restore TP53 activity in a subgroup of MPM.

In this* in vitro* approach, the effects of the nowadays state-of-the-art chemotherapeutics cisplatin and pemetrexed, alone and in combination, compared to Nutlin-3A were investigated in three cell lines covering the pattern found in patients [44, 59]. Nutlin-3A induced senescence efficiently in all three MPM cell lines and was superior compared to cisplatin and/or pemetrexed, whereas apoptosis could only be induced at high concentrations. It is known from the literature, that the effects of Nutlin-3A are cell type specific [61, 62, 64], rather inducing cell cycle arrest and senescence than apoptosis [64]. Accordingly, we investigated cisplatin and Nutlin-3A in combination to increase cellular stress by inducing platin-based DNA damage. The combination of Nutlin-3A with cisplatin results in increased apoptosis and senescence rates compared to Nutlin-3A alone, as a major function of TP53 is DNA damage and stress response [46].

The same mechanism seems to be true when combining Nutlin-3A and radiotherapy to provide additional cellular damage and shift the cellular* TP53*-response towards apoptosis, already shown in* TP53* wild-type esophageal squamous cell carcinoma* in vitro* and* in vivo* [65]. Interestingly,* Shimazu *et al. [66] found an additional growth inhibitory effect in MPM when combining Nutlin-3A with metformin, an mTOR inhibitor, suggesting a possible cross-talk between the mTOR- and TP53-pathway. Of note, the authors confirmed our findings of the cell lines NCI-H2052 and MSTO-211H as best responders to Nutlin-3A therapy, postulating an IC50 value of 0.37*μ*M (MSTO-211H) and 0.50*μ*M (NCI-H2052), respectively [66].

As mentioned before, overexpression of MDM2 can lead to a loss of P53 regulatory function via increased proteasomal degradation [39–44]. Besides its physiological inhibitor P14/ARF, analysis of the signalling relationship between these genes indicates an additional role of RB1 in this signalling network [45–51]. It has been shown, that, besides inhibition of the MDM2-TP53 interaction, Nutlin-3A also influences MDM2-RB1 interactions, making this a possible explanation for Nutlin-3A based TP53 independent effects [67].

Interestingly, even the low MDM2 expressing cell line MSTO-211H as well as the MDM2 and TP53 negative cell line NCI-H2452 shows reduced but clearly detectable, induction of apoptosis via Nutlin-3A combined with cisplatin. Also, immunohistochemically negative cells have, as reported previously [59], detectable gene expression pattern of MDM2, resulting in MDM2 protein concentrations below the detection limit of IHC. We hypothesize, as MDM2 driven regulation of TP53 is an essential mediator of apoptosis and cell state in a physiological situation, also inhibition of the TP53-MDM2 interaction at this low MDM2 levels will have a beneficial effect on cytotoxicity of platinum compounds, explaining the occurring side effects of Nutlin-3A therapy [68]. For NCI-H2452, a cell line with absent expression of TP53, the observed effect must be TP53 independently and is most likely based on RB1 inhibitory effects.

Currently, Nutlin-3A is administered* per os* as substance R05045337 in a multicentre phase I clinical trial for therapy of hematologic neoplasia [69]. Additionally, RG7112, a derivative of Nutlin-3A has entered phase I clinical trials in patients with liposarcomas that are TP53 wild-type tumors with amplified MDM2 [70]. In this clinical trial, RG7112 was administered* per os* in 20 patients in a neoadjuvant setting [68]. One patient showed partial remission and 14 showed stable disease, but all patients suffered from side effects as neutropenia [68]. A possible explanation might be the high doses of medication of 1440 mg m^−2^ day^−1^* per os* [68]. In previous* in vivo* studies, oral administration of Nutlin-3A showed several limitations as high input amounts of Nutlin-3A (200-400 mg/Kg) and difficulties in administering these high dosages [69]. It is noteworthy that efficient delivery systems were developed using polymers as poly(lactide-co-glycolide) (PLGA) and monoclonal antibodies [69].

## 5. Conclusion

In this* in vitro* study, our hypothesis that MDM2-overexpressing MPM can be targeted by a Nutlin-3A based chemotherapy was proven. Particularly, for an optimal biomarker setting of MDM2-overexpression and low/absent P14/ARF expression, superior apoptosis and senescence rates were seen compared to the conventional chemotherapeutics. Even for a less optimal biomarker setting with minimal MDM2 expression, a favorable induction of apoptosis and senescence was obvious for Nutlin-3A in combination with cisplatin compared to the conventional drug regimen. Therefore, Nutlin-3A based therapy approach could be of great value for a subgroup of patients with MPM.

## Figures and Tables

**Table 1:** Concentrations for each cytostatic substance and combination applied.

Testing Nutlin concentrations in comparison to Pemetrexed and Cisplatin	Testing Nutlin in combination with Cisplatin in comparison to Pemetrexed and Cisplatin

10nM Cisplatin	10nM Cisplatin

200nM Pemetrexed	200nM Pemetrexed

10nm Cisplatin + 200nM Pemetrexed	10nm Cisplatin + 200nM Pemetrexed

5nM Nutlin	10nm Cisplatin + 5nM Nutlin

10nM Nutlin	10nm Cisplatin + 10nM Nutlin

20nM Nutlin	10nm Cisplatin + 20nM Nutlin

	10nM Nutlin

**Table 2:** **Molecular marker constellation of the investigated MPM cell lines**. The immunoexpression or mRNA-expression of the investigated markers is shown for each investigated cell line.

Cell line	MDM2	P53	P14/ARF
NCI-H2052	“+”	“+"	“+/-”
MSTO-211H	“+/-”	“+”	“-”
NCI-H2452	“-”	“-”	“+”

-: minimal to no expression

+: expression measurable

+/-: little expression measurable

**Table:** (a) Response of MPM cell lines to pemetrexed, cisplatin, and varying Nutlin-3A concentrations

	**Cell viability**					

	10um Cis	200um Pem	10um Cis+200um Pem	5um Nut	10uM Nut	20uM Nut

H2052	+	-	+	++	+++	+++

MSTO-211H	0	+	0	0	++	+++

H2452	0	0	-	-	+	+++

	**Apoptosis**					

	10um Cis	200um Pem	10um Cis+200um Pem	5um Nut	10uM Nut	20uM Nut

H2052	0	+	0	0	0	++

MSTO-211H	0	++	+	+	0	++

H2452	0	0	0	0	++	+++

**Table:** (b) Response of MPM cell lines to varying Nutlin-3A concentrations combined with cisplatin

				**Cell viability**			

	10uM Cis	200um Pem	10um Cis+200um Pem	5um Nut+10um Cis	10uM Nut+10um Cis	20uM Nut+10um Cis	10uM Nut

H2052	0	-	0	+	++	+++	+

MSTO-211H	+	+	++	++	+++	+++	++

H2452	+	-	-	+	++	+++	+

				**Apoptosis**			

	10uM Cis	200um Pem	10um Cis+200um Pem	5um Nut+10um Cis	10uM Nut+10um Cis	20uM Nut+10um Cis	10uM Nut

H2052	0	0	0	0	+++	++	0

MSTO-211H	+	+++	+++	+	+++	++	+

H2452	+	0	+	0	++	+++	+

## Data Availability

The data used to support the findings of this study are available from the corresponding author upon request.
